# Preparation of gold nanoparticles using low-temperature heating of the dry residue of a droplet of an HAuCl_4_ solution in air

**DOI:** 10.1007/s44211-023-00438-x

**Published:** 2023-10-13

**Authors:** Kazuki Ii, Yoshiki Kurita, Naoya Kida, Shinsuke Kunimura

**Affiliations:** Department of Industrial Chemistry, Tokyo University of Science, 6-3-1 Niijuku, Katsushika-ku, Tokyo, 125-8585 Japan

**Keywords:** Gold nanoparticles, HAuCl_4_, Low cost, Low-temperature heating, Surface-enhanced Raman scattering

## Abstract

In the present study, by heating a quartz glass substrate having the dry residue of a 10 μL droplet of a solution of HAuCl_4_ and a counter substrate facing to the dry residue from room temperature to one hundred and several tens of degrees Celsius in 20 min in air, highly dense gold nanoparticles were produced on the counter substrate. A gold nanoparticle substrate produced by this simple method was utilized as a substrate for surface-enhanced Raman scattering analysis.

**Graphical abstract:**

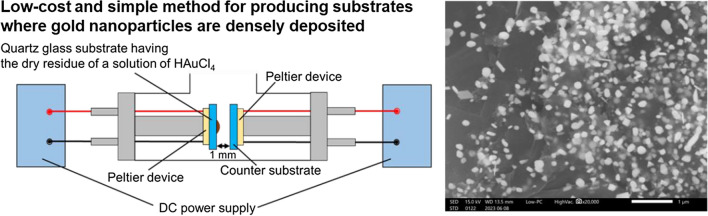

## Introduction

Using surface-enhanced Raman scattering (SERS) analysis [1–5], the detection of trace amounts of molecules can be performed. This highly sensitive analytical method would be utilized for various applications such as environmental, food, and medical analyses. Target molecules in samples are needed to be adsorbed on nanostructures of metals such as gold and silver to perform SERS analysis, and substrates having gold nanostructures are utilized for SERS analysis [6–14]. If a low-cost and simple method is employed for producing gold nanostructures on substrates, a large amount of SERS spectra would be routinely measured.

Vacuum deposition and sputtering are available for easily producing gold nanoparticle substrates. However, apparatuses for vacuum deposition and sputtering are expensive. Gold nanoparticles are also produced on substrates by heating HAuCl_4_ as described below. Palgrave et al. [15] showed that a gold thin film was formed on a substrate in a chemical vapor deposition reactor heated at 400 °C by transporting aerosols produced from a methanol solution of HAuCl_4_ to the reactor using nitrogen gas. Montero et al. [16] reported that gold nanoparticles were produced on a glassy carbon heated at 300 °C by spraying droplets of an HAuCl_4_ solution on the glassy carbon using an ultrasonic atomizer. Chen et al. [17] showed that by heating an ethanol solution of HAuCl_4_ and a substrate placed away from the solution at 500 °C for 2 h under a pressure of 600 mTorr while flowing nitrogen gas, gold nanoparticles were formed on the substrate, and they explained the production mechanism as follows:

1. Gaseous AuCl and AuCl_3_ occurred by heating HAuCl_4_ are transported to the substrate by nitrogen gas.
2. A film of a mixture of AuCl and AuCl_3_ attributed to gaseous AuCl and AuCl_3_ is formed on the substrate.
3. The production of metallic gold seeds from the mixture of AuCl and AuCl_3_ and the growth of metallic gold seeds are caused by heating

According to a supporting information of the paper reported by Chen et al. [17], the formation of gold nanoparticles was observed in a scanning electron microscope (SEM) image of the product produced on a substrate by heating the substrate and a solution of HAuCl_4_ placed away from the substrate at 100 °C at atmospheric pressure. This result indicated that the production of gaseous gold chloride species from HAuCl_4_ and the production of gold nanoparticles originating from the gaseous products occurred even when the heating temperature was 100 °C, but the rate for the production of metallic gold seeds and the growth rate of gold nanocrystals were shown to be slow at such a low heating temperature.

Recently, our research group reported that gold nanoparticles with high purity were produced on a counter substrate when a quartz glass substrate having the dry residue of a 10 µL droplet of a solution of HAuCl_4_ and the counter substrate facing to the dry residue were simultaneously heated from room temperature to one hundred and several tens of degrees Celsius in 9 min in a low vacuum and a gold nanoparticle substrate produced by this method was utilized as a SERS substrate for the detection of nicotinamide [18]. The production mechanism of gold nanoparticles produced by this method would be similar to that reported by Chen et al. [17]. However, our research group showed that the rate for the production of metallic gold from gold chloride species and the growth rate for metallic gold seeds would be fast enough at such a low heating temperature in a low vacuum because highly pure gold nanoparticles were produced by this method. This method for producing gold nanoparticles has the following advantages: (1) the cost for constructing an apparatus for producing gold nanoparticles is low; (2) the procedure in this method is simple; (3) a carrier gas is not used during the production of gold nanoparticles; and (4) waste liquid does not occur after the production of gold nanoparticles. The procedure in this method would become simpler if the production is performed in air. In the present study, a substrate having the dry residue of a droplet of a solution of HAuCl_4_ and a counter substrate were simultaneously heated from room temperature to one hundred and several tens of degrees Celsius in 20 min in air to prepare gold nanoparticles on the counter substrate. We show that gold nanoparticle substrates that are available for SERS analysis would be produced by this low-cost and simple production method.

## Experimental

**Fig. 1:**
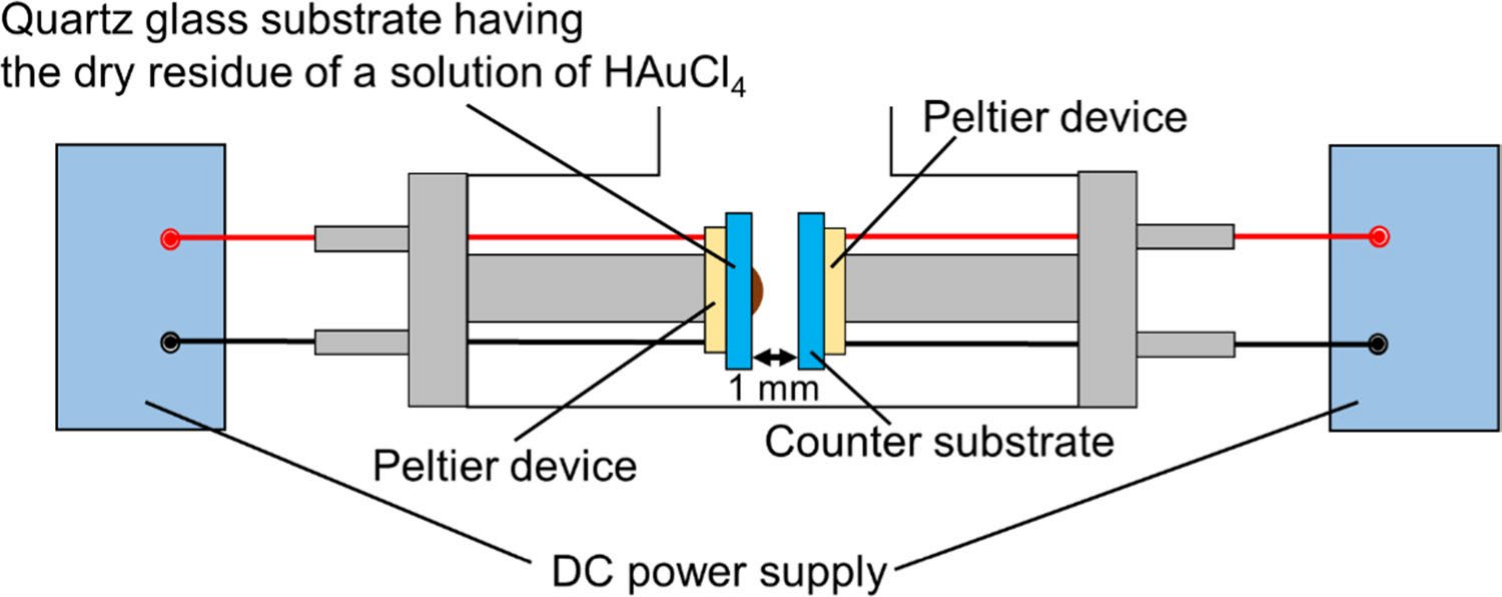
Schematic view of an apparatus for producing gold nanoparticles

Figure 1 shows a schematic view of an apparatus for producing gold nanoparticles in the present study. A 10 μL droplet of a commercially available 1000 mg L^−1^ gold standard solution (FUJIFILM Wako Pure Chemical Co., Osaka, Japan) was dried on a quartz glass substrate in air. This gold standard solution is an HCl acidic solution of HAuCl_4_·4H_2_O. The quartz glass substrate where the dry residue of the HAuCl_4_ solution was placed and a counter substrate were placed on Peltier devices, and these substrates faced each other with a distance of about 1 mm in a container. A DC current of 1.2 A was applied to the Peltier devices to increase temperatures of the substrate having the dry residue of the HAuCl_4_ solution and the counter substrate, and these two substrates were simultaneously heated using the Peltier devices for 20 min in air. When one of the two Peltier devices and a quartz glass substrate attached on the other Peltier device were arranged so as to face each other in the container and a DC current of 1.2 A was applied to these Peltier devices for 20 min in air, the temperature of the surface of the Peltier device on which no quartz glass substrate was placed, which was measured using a chromel–alumel thermocouple, increased from room temperature to about 180 °C in 20 min. Quartz glass substrates with a diameter of 20 mm and a thickness of 1 mm were used in the present study. In the present paper, the procedure described above is called Procedure A. In the present study, another procedure, which was similar to Procedure A, was also conducted, and this procedure is called Procedure B. In Procedure B, a quartz glass substrate having the dry residue of a 10 μL droplet of the HAuCl_4_ solution was heated by the Peltier device for 20 min in air, but heating a counter substrate was not performed.

Raman spectra were measured by a portable Raman spectrometer (C12710, Hamamatsu Photonics K. K., Hamamatsu, Japan). A droplet of a sample solution was irradiated with the laser beam with a wavelength of 785 nm during the measurement, and a Raman spectrum was obtained by averaging five spectra, which were measured for 10 s, respectively. This Raman spectrometer was used in “High” mode. The laser power is 50 mW when a typical C12710 spectrometer is used in this mode. Total reflection X-ray fluorescence (TXRF) spectra were measured in air for 600 s using a portable TXRF spectrometer [19–21], and the setup of the portable TXRF spectrometer employed in the present study was the same as those reported elsewhere. [22] A tantalum anode X-ray tube was operated at 25 kV and 0.2 mA. An SEM (JCM-7000 NeoScope, JEOL Ltd., Akishima, Japan) was used for acquiring secondary electron images, and the acceleration voltage of the electron beam was set to 15 kV.

## Results and discussion

**Fig. 2:**
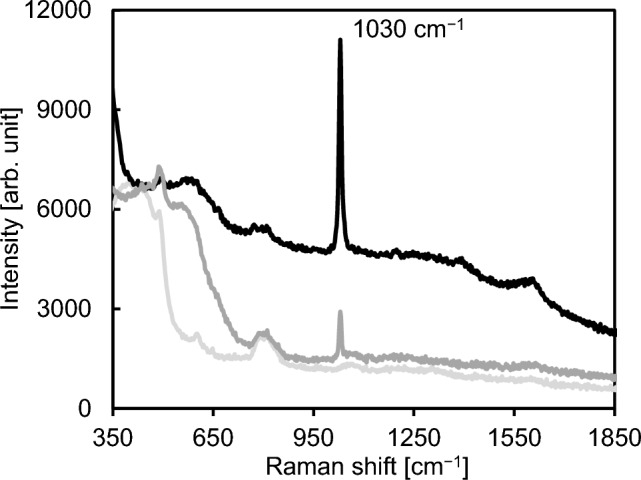
Representative Raman spectra of 5 μL droplets of a 0.1 g L^−1^ nicotinamide solution, which were placed on a quartz glass substrate (light gray line), the product produced on a quartz glass substrate used as a counter substrate by Procedure A (black line), and the product produced on a quartz glass substrate used as a counter substrate by Procedure B (dark gray line)

**Fig. 3:**
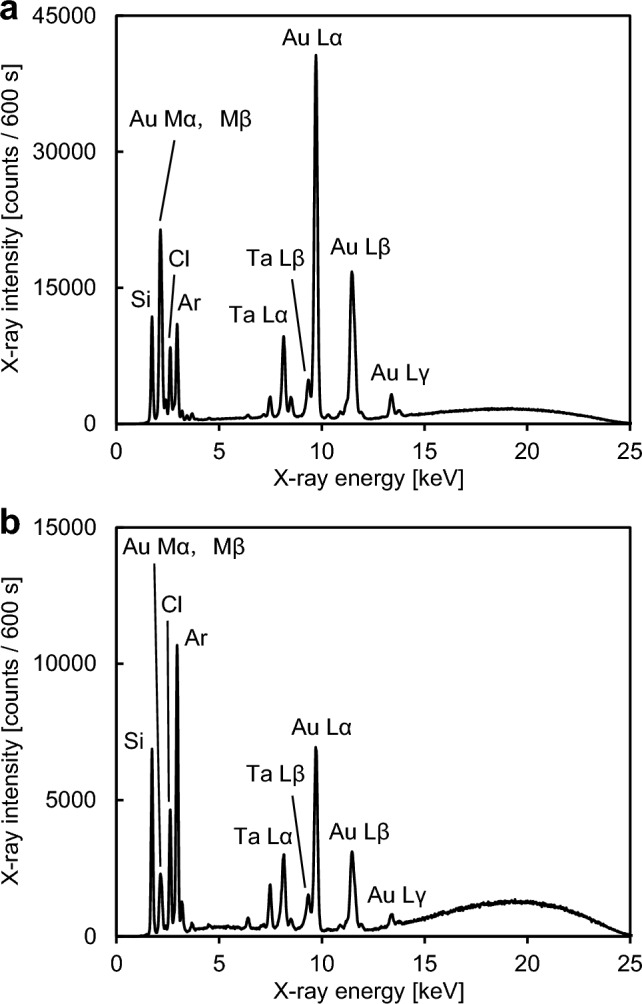
Representative TXRF spectra of products produced on quartz glass substrates used as counter substrates by **a** Procedure A and **b** Procedure B

**Fig. 4:**
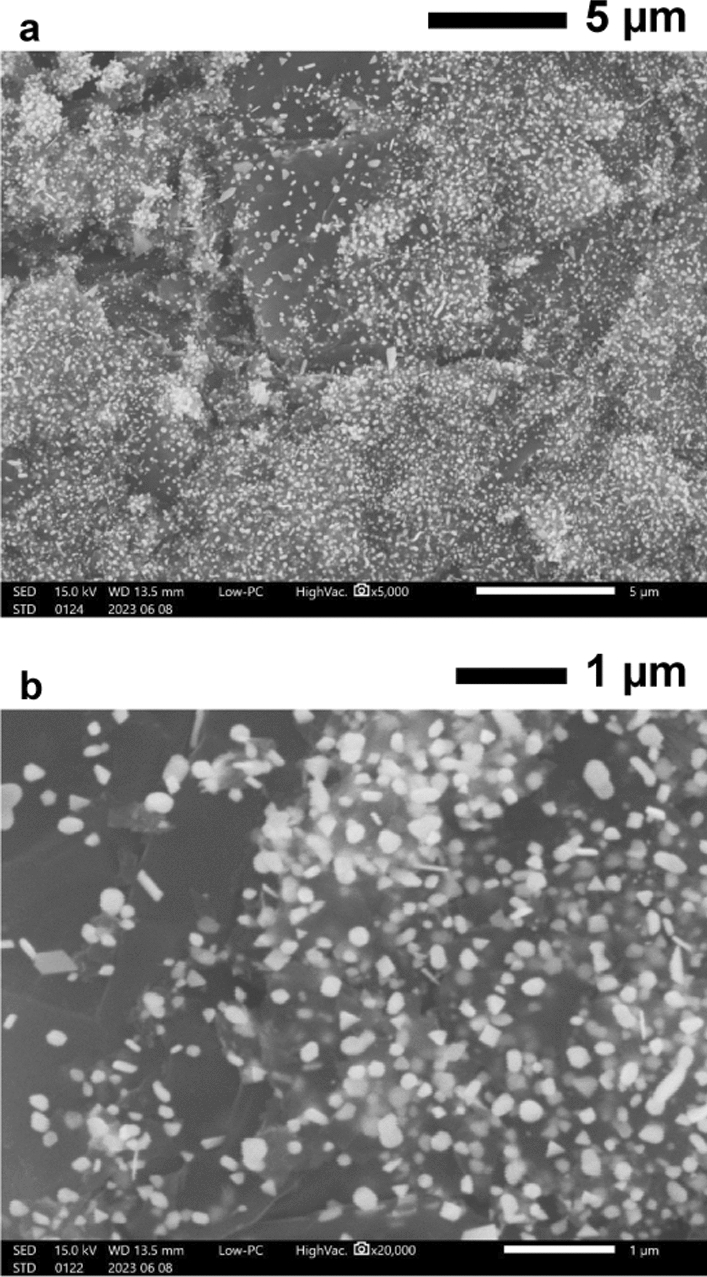
Secondary electron images of the product containing gold nanoparticles produced on a carbon sheet used as a counter substrate by Procedure A at **a** ×5000 magnification and **b** ×20,000 magnification

Figure 2 shows representative Raman spectra of 5 µL droplets of a 0.1 g L^−1^ nicotinamide solution, which were placed on a quartz glass substrate, the product produced by Procedure A, and the product produced by Procedure B. These products were produced on quartz glass substrates that were used as counter substrates. The Raman spectra of the sample droplets on these products were measured on the day when they were produced. Pal et al. [23] reported that a strong Raman peak was observed at 1032 cm^−1^ when the SERS spectrum of a nicotinamide solution on a silver-coated alumina substrate was measured, and SERS spectra of nicotinamide in the concentration ranging from 10^−2^ to 10^−6^ mol L^−1^ were measured in their study. As shown in Fig. 2, a Raman peak attributed to nicotinamide was observed at 1030 cm^−1^ when the sample droplets on the products produced by Procedure A and Procedure B were measured, and this Raman peak did not appear in the spectrum of the sample droplet on the quartz glass substrate. Intensities of Raman peaks originating from molecules adsorbed on gold nanostructures would be enhanced due to SERS effect. Using the products produced by Procedure A and Procedure B as sample holders enhanced the intensity of the Raman peak attributed to nicotinamide compared with the use of the quartz glass substrate, and this result showed that gold nanoparticles were contained in these products. However, the intensity of the Raman peak originating from nicotinamide obtained with the product produced by Procedure B was lower than that obtained with the product produced by Procedure A. Therefore, Procedure A was more effective for increasing the amount of metallic gold nanoparticles having suitable size for SERS analysis than Procedure B. Because the strong Raman peak originating from 0.1 g L^−1^ of nicotinamide, which was calculated to be equivalent to 8 × 10^−4^ mol L^−1^ of nicotinamide, was observed in the spectrum obtained with the product produced by Procedure A, the use of this product would enable the detection of a few tens of milligrams per liter of nicotinamide. The detection sensitivity obtained with the gold nanoparticles produced by Procedure A would be somewhat worse than that in the previous paper reported by Pal et al. [23]. However, optimizing production conditions for gold nanoparticles would lead to an improvement in the detection sensitivity. Figure 3 shows representative TXRF spectra of products produced by Procedure A and Procedure B. These two products were produced on quartz glass substrates used as counter substrates. These spectra were measured on the day when these products were produced. As shown in Fig. 3, the Au M lines, the Au L lines, and the Cl Kα line were observed. The Cl peak in Fig. 3 would originate from solid gold chloride species such as AuCl_3_ on the counter substrate, which were attributed to gaseous gold chloride species produced by heating the dry residue of the HAuCl_4_ solution. The ratio of the net intensity of the Cl Kα peak to the sum of net intensities of Au Mα and Mβ peaks in the spectrum of the product produced by Procedure A and that in the spectrum of the product produced by Procedure B were 0.23 and 1.32, respectively. This result showed that Procedure A was more effective for enhancing the ratio of the amount of metallic gold to that of gold chloride species than Procedure B. The Si, Ar, and Ta peaks in Fig. 3 were attributed to the quartz glass substrate, argon contained in air, and the anode material of the X-ray tube, respectively. Figure 4 shows secondary electron images of the product containing gold nanoparticles produced on a carbon sheet by Procedure A. This carbon sheet was attached on a quartz glass substrate and was used as a counter substrate. These SEM images were obtained on the day when the product containing gold nanoparticles was produced. In a previous study [18], when gold nanoparticles, which were produced on a carbon sheet by simultaneously heating a quartz glass substrate having the dry residue of an HAuCl_4_ solution and the carbon sheet from room temperature to one hundred and several tens of degrees Celsius in 9 min in a low vacuum, were observed and analyzed by SEM equipped with an energy-dispersive X-ray spectrometer, nanoparticles in SEM images were metallic gold and particulate gold chloride species were not observed. Therefore, almost all nanoparticles observed in Fig. 4 were concluded to be metallic gold although the product on the carbon sheet would be consisted of metallic gold nanoparticles and remaining gold chloride species as shown in Figs. 2 and 3. As shown in Fig. 4a, gold nanoparticles were densely distributed on the carbon sheet. Figure 4b shows that gold nanoparticles with the sizes from several tens of nanometers to about 200 nm were mainly produced.

In the previous paper [18], the ratio of the amount of metallic gold to that of gold chloride species in the product produced on a counter substrate by simultaneously heating a quartz glass substrate having the dry residue of an HAuCl_4_ solution and the counter substrate in a low vacuum was higher than that in the product on a counter substrate produced by heating only a quartz glass substrate where the dry residue of the HAuCl_4_ solution was placed in a low vacuum. As shown Figs. 2 and 3, the present study indicated that Procedure A was more effective for increasing the amount of gold nanoparticles having suitable size for SERS analysis and the ratio of the amount of metallic gold to that of gold chloride species in the product than Procedure B. These results in present study were similar to those that were described above and were shown in the previous paper [18]. According to a supporting information of the paper reported by Chen et al. [17], gaseous gold chloride species were produced from HAuCl_4_ and gold nanoparticles originating from the gaseous products were formed on a substrate placed away from a solution of HAuCl_4_ even when the heating temperature was 100 °C. However, in the paper reported by Chen et al.[17], the rate for the production of metallic gold from a film of a mixture of AuCl and AuCl_3_ and the growth rate of gold nanocrystals were shown to be slow at such a low heating temperature. On the other hand, in the present study, highly dense gold nanoparticles were produced on a counter substrate by increasing temperatures of a substrate having the dry residue of the HAuCl_4_ solution and the counter substrate from room temperature to one hundred and several tens of degrees Celsius in 20 min in air. The production of gold nanoparticles was performed without using a carrier gas. The use of a gold nanoparticle substrate produced by this production method led to a significant enhancement in the intensity of a Raman peak originating from nicotinamide. The present study suggests the following points:

1. Gaseous gold chloride species produced by heating the dry residue of an HAuCl_4_ solution in air reach a counter substrate without using a carrier gas, and solid gold chloride species originating from the gaseous gold chloride species are deposited on the counter substrate.
2. The rate for the production of gold nanoparticles from the solid gold chloride species is fast enough even when the counter substrate is heated at such a low heating temperature for a short time in air. Therefore, a highly dense gold nanoparticle substrate was produced using this method.

## Conclusions

Highly dense gold nanoparticles are produced on a counter substrate by performing simultaneous low-temperature heating of a quartz glass substrate having the dry residue of a droplet of a solution of HAuCl_4_ and a counter substrate for 20 min in air. This low-cost and simple production method is beneficial for preparing SERS substrates.

## Data Availability

The raw data used for preparing figures in this paper are available from the corresponding author on reasonable request.
